# Can Aidi injection restore cellular immunity and improve clinical efficacy in non-small-cell lung cancer patients treated with platinum-based chemotherapy? A meta-analysis of 17 randomized controlled trials following the PRISMA guidelines

**DOI:** 10.1097/MD.0000000000005210

**Published:** 2016-11-04

**Authors:** Zheng Xiao, Chengqiong Wang, Yongping Sun, Nana Li, Jing Li, Ling Chen, Xingsheng Yao, Jie Ding, Hu Ma

**Affiliations:** a Evidence-based Medicine Center, MOE Virtual Research Center of Evidence-based Medicine at Zunyi Medical College; b Department of Respiratory Medicine (Center for Evidence-based and Translational Medicine of Major Infectious Diseases), Affiliated Hospital of Zunyi Medical College, Zunyi 563000, Guizhou, China; c Teaching and Research Group of Evidence-based Medicine, Zhuhai Campus of Zunyi Medical College, Zhuhai, Guangdong, China; d Department of Immunology, Zunyi Medical College; e Department of Oncology, Affiliated Hospital of Zunyi Medical College; f Department of Neurology, First People's Hospital of Zunyi City and Third Affiliated Hospital of Zunyi Medical College; g Outpatient Department of Psychological Counseling Clinic (Center for Evidence-based and Translational Medicine of Major Infectious Diseases), Affiliated Hospital of Zunyi Medical College, Zunyi, Guizhou, China.

**Keywords:** adjuvant chemotherapy, Aidi injection, cellular immunity, meta-analysis, non-small-cell lung cancer, platinum-based chemotherapy

## Abstract

**Background:**

Aidi injection is an adjuvant chemotherapy drug commonly used in China. Can Aidi injection restore the cellular immunity and improve the clinical efficacy in non-small-cell lung cancer (NSCLC) patients treated with platinum-based chemotherapy? There is a lack of strong evidence to prove it. To further reveal it, we systematically evaluated all related studies. We collected all studies about the clinical efficacy and cellular immunity of Aidi injection plus platinum-based chemotherapy for NSCLC in Medline, Embase, Web of Science, China national knowledge infrastructure database (CNKI), Chinese Scientific Journals Full-Text Database (VIP), Wanfang, China biological medicine database (CBM) (established to June 2015), Cochrane Central Register of Controlled Trials (CCRCT) (June 2015), Chinese clinical trial registry, and US-clinical trials (June 2015). We evaluated their quality according to the Cochrane evaluation handbook of randomized controlled trials (RCTs) (5.1.0), extracted data following the patient intervention control group outcomes principles and synthesized the data by meta-analysis. Seventeen (RCTs) with 1390 NSCLC patients were included, with general methodological quality in most trials. The merged relative risk (RR) values and their 95% CI of meta-analysis for objective response rate (ORR) and disease control rate (DCR) were as follows: 1.26 (1.12, 1.42) and 1.11(1.04, 1.17). The merged standardized mean difference (SMD) values and their 95% CI of meta-analysis for the percentage of CD3^+^T cells, CD4^+^T cells, CD8^+^T cells, natural killer (NK) cells, and CD4^+^/CD8^+^ T cell ratio were as follows: 1.41, (0.89, 1.92), 1.59, (1.07, 2.11), 0.85, (0.38, 1.33), 1.64 (0.89, 2.39) and 0.91, (0.58, 1.24). Compared with platinum-based chemotherapy alone, all differences were statistically significant. These results might be overestimated or underestimated.

**Conclusions:**

Aidi injection plus platinum-based chemotherapy can improve the clinical efficacy of patients with NSCLC. Aidi injection could significantly restore the cellular immunity damaged by platinum-based chemotherapy. It may be an important tumor immune modulator and protector for patients with NSCLC treated with chemotherapy.

## 1 Introduction

Lung cancer is the leading cause of cancer death in both more and less developed countries.^[1–2]^ Approximately 80% of lung cancers are non-small-cell lung cancers (NSCLC). Over 50% of patients with NSCLC have advanced local invasion and metastasis and therefore lose the opportunity for surgery.^[3]^ Hence, they are forced to accept the systemic chemotherapy, radiotherapy, or chemoradiotherapy.^[4–5]^ Platinum-based chemotherapy is an important treatment strategy for advanced stage and metastasis of NSCLC.^[6–7]^ The systemic chemotherapy can damage the host immune cells and impair the antitumor response.^[8–9]^ All these lead to poor clinical efficacy and substandard quality of life (QOL) for patients. Therefore, finding a way to restore host immunity and improve clinical efficacy is consequential.

Aidi injection (Z52020236, China food and Drug Administration) is an adjuvant chemotherapy drug commonly used in China, which is composed by the extracts of *Astragalus* (*Astragalus membranaceus*), *Eleutherococcus senticosus* (*Acanthopanax senticosus*), ginseng (*Panax ginseng* C. A. Mey), and cantharidin (*Lytta vesicatoria*). Most studies^[10–13]^ had shown that *Astragalus*, *Eleutherococcus senticosus*, cantharidin, and ginseng appear to improve host immunity through relieving immunosurveillance and restoring T-cell function damaged by chemotherapy. Can Aidi injection restore the cellular immunity and improve the clinical efficacy in NSCLC patients receiving platinum-based chemotherapy? Related studies ^[14–15]^ had shown that Aidi injection might restore cellular immunity and improve the clinical efficacy in NSCLC patients. Unfortunately, these conclusions were different in different studies with lower sample size. There is a lack of strong evidence to prove it. To reveal whether Aidi injection can restore the cellular immunity and improve the clinical efficacy in NSCLC patients receiving platinum-based chemotherapy, we systematically evaluated all related studies.

## 2 Methods

This article followed Preferred Reporting Items for Systematic Reviews and Meta-Analyses (PRISMA) guidelines.

### 2.1 Literature search strategy

Two reviewers (Cheng-qiong Wang and Yongping Sun) independently searched articles in electronic databases using the search strategy (aidi or aidi injection) and (“Lung neoplasms” [Mesh] or pulmonary neoplasms or lung neoplasm or pulmonary neoplasm or lung cancer or lung cancers or pulmonary cancer or pulmonary cancers or lung carcinoma or pulmonary carcinoma or NSCLC). The published studies were retrieved in Medline, Embase, Web of Science, China National Knowledge Infrastructure Database (CNKI), Chinese Scientific Journals Full-Text Database (VIP), Wanfang Database, China Biological Medicine Database (CBM) (established to June 2015), and Cochrane Central Register of Controlled Trials (CCRCT, Issue 6 of 12, June 2015). Unpublished studies were retrieved in Chinese clinical trial registry and US-clinical trials (established to June 2015). All retrievals were implemented by the Mesh and free word. No language restrictions were placed on the search. Ethical approval was not required, as our study is a meta-analysis of published studies.

### 2.2 Studies inclusion and exclusion criteria

#### 2.2.1 Inclusion criteria

Included studies must meet the following criteria: the disease was diagnosed and confirmed with NSCLC (III-IV stage) in accordance with histopathological and cytological diagnostic criteria; there were randomized controlled trials (RCTs); the experimental group was Aidi injection plus platinum-based chemotherapy including vinorelbine and cisplatin (NP), cisplatin and docetaxel (DP), or cisplatin and gemcitabine (GP), control group was platinum-based chemotherapy alone; subjects before being included in the study did not receive other therapies including other Chinese herbs and intra-arterial infusion chemotherapy; there were no severe damages in liver or kidney function in any of the patients; according to the World Health Organization (WHO) guidelines^[16]^ for solid tumor responses, indicators were complete response (CR), partial response (PR), no change (NC), progressive disease (PD), objective response rate (ORR) equals CR + PR and disease control rate (DCR) equals CR + PR + NC, clinical efficacy was evaluated by objective response rate (ORR) and disease control rate (DCR), cellular immunity was evaluated by the percentage of CD3^+^T cells, CD4^+^T cells, CD8^+^T cells and natural killer cells (NK cells) and the CD4^+^/CD8^+^ T cells ratio in peripheral blood, all cells were detected with immunocytochemistry or flow cytometry; time and settings: no restrictions were set on the duration of follow-ups or types of settings.

#### 2.2.2 Exclusion criteria

Excluded studies must meet the following criteria: duplicated articles; unrelated studies including other themes, and animal and in-vitro studies; nonrandomized controlled studies; abstracts and reviews without specific data; studies with inaccurate information or non-usable statistical data.

### 2.3 Study quality evaluation

We evaluated the quality of all included studies according to the Cochrane evaluation handbook of RCTs (5.1.0).^[17]^ The bias parameters were the random sequence generation (selection bias), the allocation concealment (selection bias), the blinding of participants and personnel (performance bias), the blinding of outcome assessment (detection bias), the incomplete outcome data (attrition bias), the selective report (reporting bias), and the other bias. We judged each item on 3 levels (“Yes” for low bias, “No” for high risk of bias and “Unclear”). Then, we assessed the trials and categorized them into three levels: low risk of bias (all the items were categorized “Yes”), high risk of bias (at least one item ranked “No”) and unclear risk of bias (at least one item was “Unclear”).

### 2.4 Selection and evaluation of articles

Two reviewers (Jing Li and Cheng-qiong Wang) independently selected and evaluated articles according to the above standards. Any disagreements were resolved by discussion between themselves or with Xingsheng Yao.

### 2.5 Data extraction and statistical analysis

Two reviewers (Nana Li and Cheng-qiong Wang) independently extracted all data including: publishing time and country; study design including the randomization methods, demographic characteristics and blinding implementation; the sample size of experimental and control group, clinical efficacy and cellular immunity. Meta-analysis was done by two reviewers (Jing Li and Zheng Xiao) with Review Manager 5.3 (The Cochrane Collaboration, Oxford, UK). The relative risk (RR), standardized mean difference (SMD) and 95% confidence intervals (CI) were calculated. Statistical heterogeneity across trials was assessed by χ^2^-based Q-statistic test and the consistency was calculated by *I*^2^. If the homogeneity (*P* ≥ 0.1, *I*^2^ ≤ 50%) was not rejected, the fixed-effects model was used to calculate the summary RR or SMD and the 95% CI. The results were analyzed by random-effects model if the heterogeneity (*P* < 0.1, *I*^2^ > 50%) was higher and the results of the fixed-and random-effect model had good consistency. The clinical heterogeneity was handled by strict inclusion and exclusion criteria and subgroup analysis. Statistical heterogeneity was reduced by random-effects model if the results of the fixed- and random-effect model had good consistency. Otherwise, the results were analyzed by descriptive analysis. Publication bias was evaluated through funnel plots if there were more than 10 included studies. The sensitivity was evaluated through deleting the studies with high weight and significant differences.

## 3 Results

### 3.1 Search results

**Figure 1:**
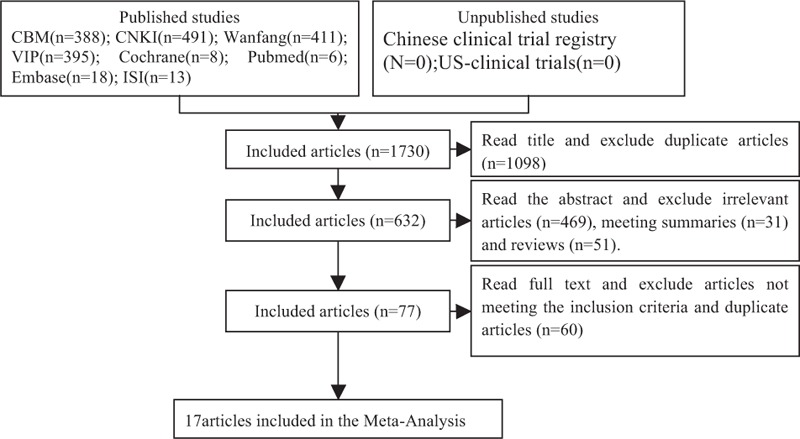
Articles retrieved and assessed for eligibility. After successively applying the study exclusion criteria, 17 randomized controlled trials (RCTs) were included.

The initial database search identified 1730 published studies using our search strategies and unpublished studies were not retrieved (Fig. 1). After successively applying the study exclusion criteria, 17 RCTs were included.

### 3.2 Characteristics of the included studies

Seventeen RCTs with 1390 NSCLC patients (III-IV stage) in China were included in this meta-analysis (Table 1). The cases of Aidi injection plus platinum-based chemotherapy and chemotherapy alone were 701 and 689, respectively. The males and females were 814 and 460, respectively, with age range between 27 and 78 years. The dosage of Aidi injection was 40–100 mL/day and treatment time was 10 days to 28 days. Clinical efficacy and cellular immunity were evaluated at 2 weeks to 12 weeks.

**Table 1:**
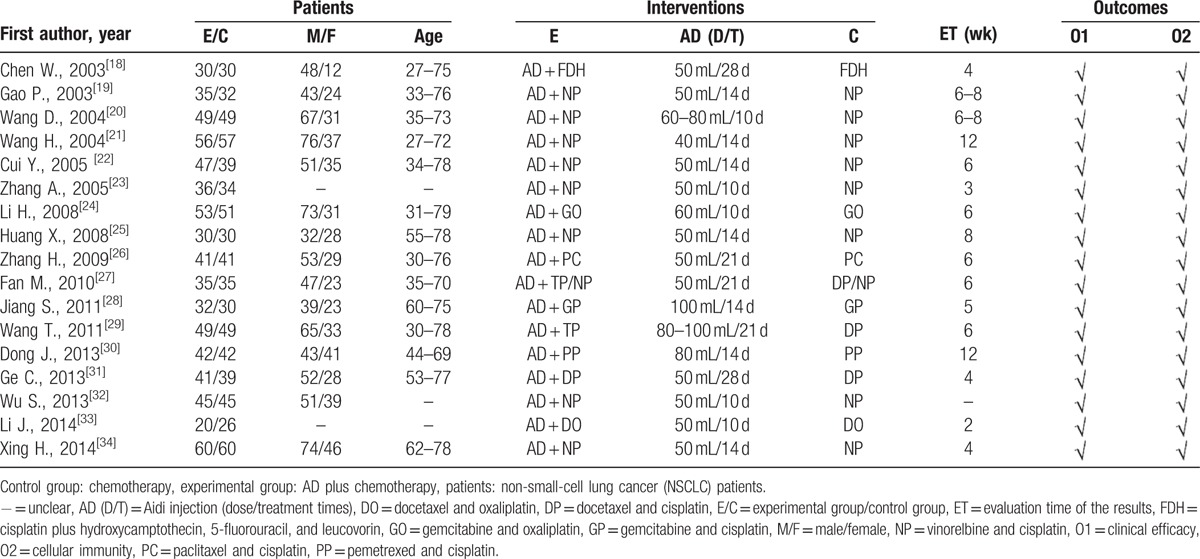
The characteristics of the included studies.

### 3.3 Methodological bias of the included studies

**Figure 2:**
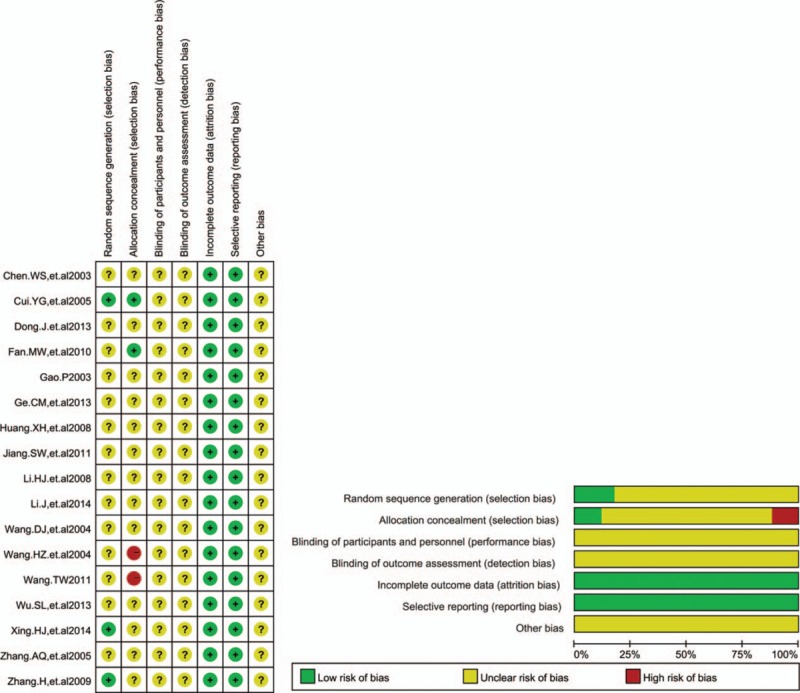
Risk of methodological bias of the included studies. There was general methodological quality in most trials.

In 17 trials, the methods of random allocation were described clearly in only 3 trials. This indicated that there was selectivity bias in the included studies. The random allocation concealment was implemented by envelope and was open by hospitalization orders respectively in 2 trials. Not all the included studies were described as blinding to patients and doctors. Therefore, it indicated that there was selection bias. All data were complete and selective report did not appear in all of the studies. Other bias was not clear. Characteristics and quality of all included studies are presented in Figure 2.

### 3.4 Clinical efficacy

**Figure 3:**
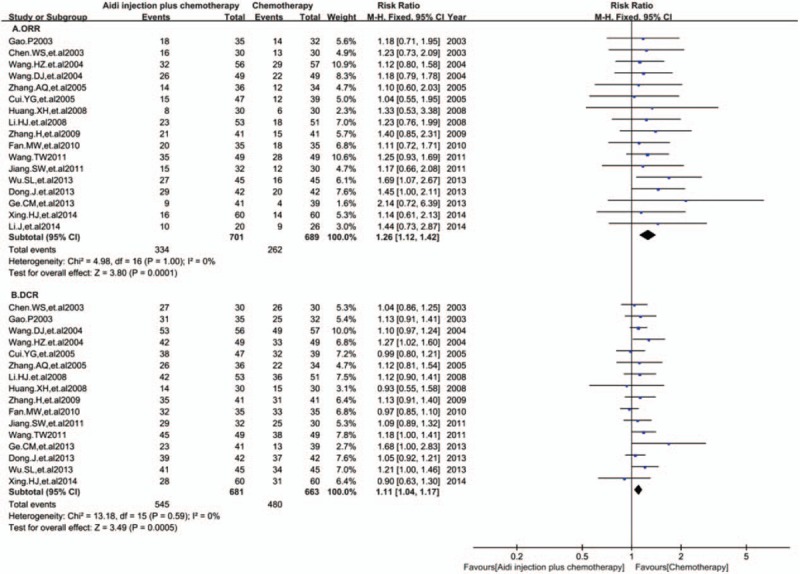
(A) Meta-analysis of the ORR between 2 groups. Meta-analysis showed that the ORR was statistically different between the 2 groups [RR = 1.26, 95% CI (1.12, 1.42), *P* = 0.0001] by fixed-effects model. (B) Meta-analysis of the DCR between 2 groups. Meta-analysis showed that the DCR was statistically different between the 2 groups [RR = 1.11, 95% CI (1.04, 1.17), *P* = 0.0001] by fixed-effects model. DCR = disease control rate, ORR = objective response rate, RR = relative risk.

Seventeen RCTs with 1390 cases were included (Fig. 3A). There was homogeneity between studies (*I*^2^ = 0%). Meta-analysis showed that the ORR was statistically different between the 2 groups [RR = 1.26, 95% CI (1.12, 1.42), *P* = 0.0001] by fixed-effects model. Sixteen RCTs with 1344 cases were included (Fig. 3B). There was homogeneity between studies (*I*^2^ = 0%). Meta-analysis showed that the DCR was statistically different between the 2 groups [RR = 1.11, 95% CI (1.04, 1.17), *P* = 0.0005] by fixed-effects model. All results showed that compared with chemotherapy alone, Aidi injection plus platinum-based chemotherapy could significantly improve the ORR and DCR of patients with NSCLC.

### 3.5 Cellular immunity

#### 3.5.1 CD3^+^T cells

**Figure 4:**
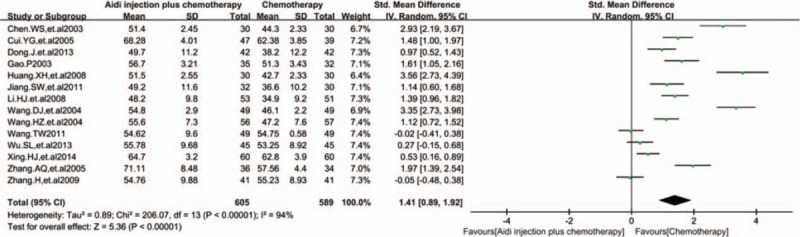
Meta-analysis of the CD3^+^T cells. Meta-analysis showed that the percentage of CD3^+^T cells was statistically different between the 2 groups [SMD = 1.41, 95% CI (0.89, 1.92), *P*<0.00001] by random effect model. SMD = standardized mean difference.

In 17 RCTs, 14 trials with 1194 cases were included (Fig. 4). There was statistical heterogeneity between studies (*I*^2^ = 94%). Meta-analysis showed that the percentage of CD3^+^T cells was statistically different between the 2 groups [SMD = 1.41, 95% CI (0.89, 1.92), *P* < 0.00001] by random-effect model.

#### 3.5.2 CD4^+^ T cells

**Figure 5:**
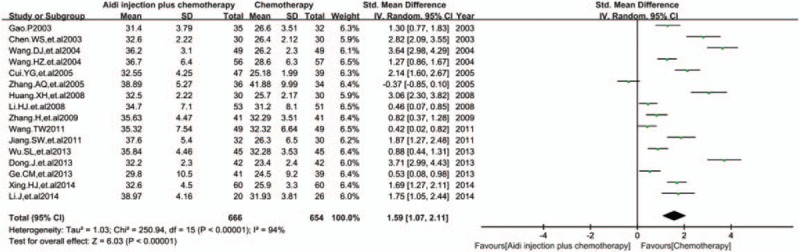
Meta-analysis of the CD4^+^T cells. Meta-analysis showed that the percentage of CD4^+^ T cells was statistically different between the 2 groups [SMD = 1.59, 95% CI (1.07, 2.11), *P*<0.00001] by random effect model. SMD = standardized mean difference.

In 17 RCTs, 16 trials with 1320 cases were included (Fig. 5). There was statistical heterogeneity between studies (*I*^2^ = 94%). Meta-analysis showed that the percentage of CD4^+^ T cells was statistically different between the 2 groups [SMD = 1.59, 95% CI (1.07, 2.11), *P* < 0.00001] by random-effect model.

#### 3.5.3 CD8^+^T cells

**Figure 6:**
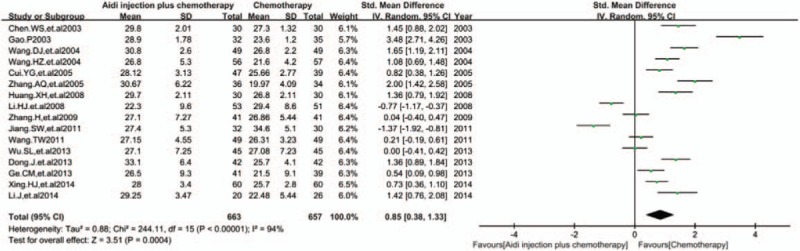
Meta-analysis of CD8^+^T cells. Meta-analysis showed that the percentage of CD8^+^T cells was statistically different between the 2 groups [SMD = 0.85, 95% CI (0.38, 1.33), *P* = 0.0004] by random effect model. SMD = standardized mean difference.

In 17 RCTs, 16 trials with 1320 cases were included (Fig. 6). There was statistical heterogeneity between studies (*I*^2^ = 94%). Meta-analysis showed that the percentage of CD8^+^T cells was statistically different between the 2 groups [SMD = 0.85, 95% CI (0.38, 1.33), *P* = 0.0004] by random-effect model.

#### 3.5.4 CD4^+^/CD8^+^ T cells ratio

**Figure 7:**
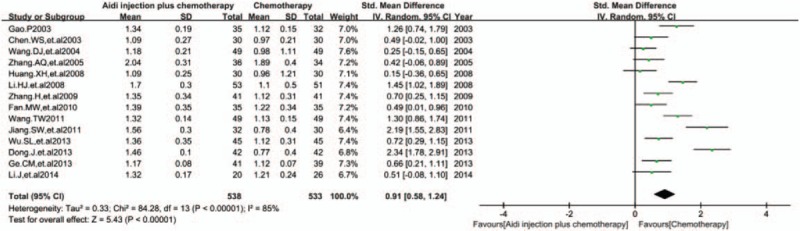
Meta-analysis of CD4^+^/CD8^+^ T cells ratio. Meta-analysis showed that the CD4^+^/CD8^+^ T cells ratio was statistically different between the 2 groups [SMD = 0.91, 95% CI (0.58, 1.24), *P*<0.00001] by random effect model. SMD = standardized mean difference.

In 17 RCTs, 14 trials with 1071 cases were included (Fig. 7). There was statistical heterogeneity between studies (*I*^2^ = 85%). Meta-analysis showed that the CD4^+^/CD8^+^ T cells ratio was statistically different between the 2 groups [SMD = 0.91, 95% CI (0.58, 1.24), *P* < 0.00001] by random-effect model.

### 3.6 NK cells

**Figure 8:**
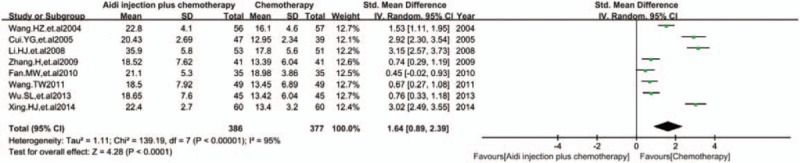
Meta-analysis of NK cells. Meta-analysis showed that the percentage of NK cells was statistically different between the 2 groups [SMD = 1.64, 95% CI (0.89, 2.39), *P*<0.0001] by random effect model. NK = natural killer, SMD = standardized mean difference.

In 17 RCTs, 8 trials with 763 cases were included (Fig. 8). There was statistical heterogeneity between studies (*I*^2^ = 95%). Meta-analysis showed that the percentage of NK cells was statistically different between the 2 groups [SMD = 1.64, 95% CI (0.89, 2.39), *P* < 0.0001] by random-effect model. All results showed that Aidi injection could significantly improve the percentage of CD3^+^ T cells, CD4^+^ T cells, CD8^+^ T cells, and NK cells, and the CD4^+^/CD8^+^ T cells ratio in the peripheral blood of patients with NSCLC.

### 3.7 Publication bias and sensitivity analysis

**Figure 9:**
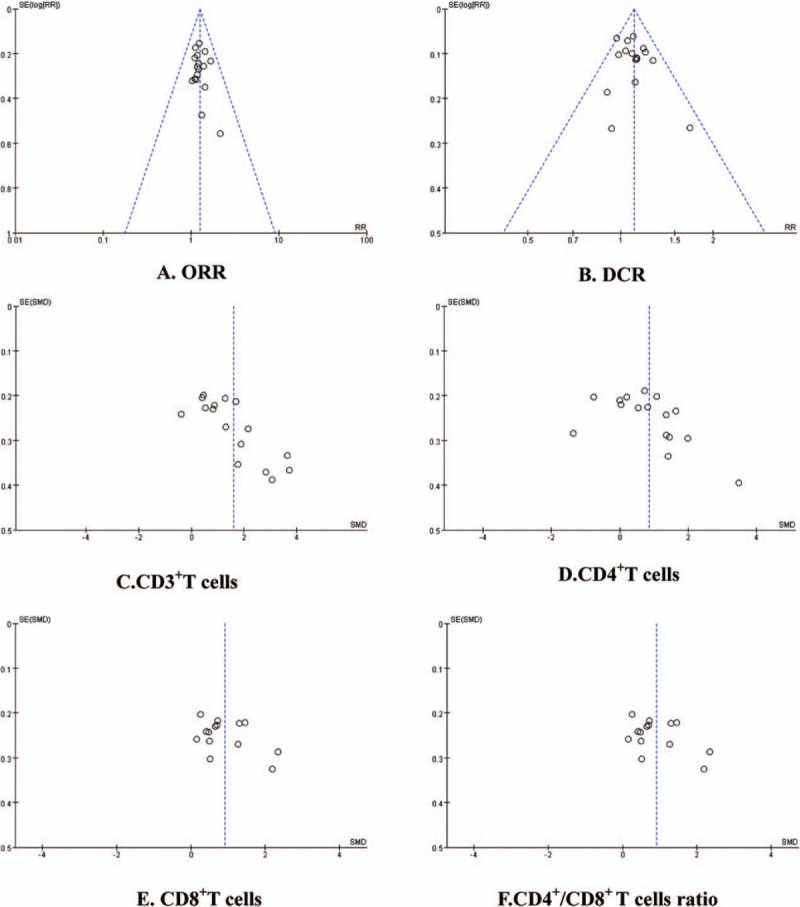
Funnel plot. There was a publication bias in included studies which influenced the results of our analysis.

The funnel plots were symmetric in the studies about ORR and DCR (Fig. 9A and B). This indicated that there was no publication bias in these studies which objectively reported the results. The funnel plots were significantly asymmetric in the studies about the CD3^+^T cells, CD4^+^T cells, CD8^+^T cells, and CD4^+^/CD8^+^ T cells ratio (Fig. 9C–F). Results showed that all points were asymmetric and some points were distributed outside of the funnel. This indicated that there was publication bias in the included studies which influenced the results of our analysis. In all, these results might be overestimated or underestimated. After excluding the over- or underestimated studies, meta-analysis showed that the results before and after exclusion had a good consistency (Table 2).

**Table 2:**
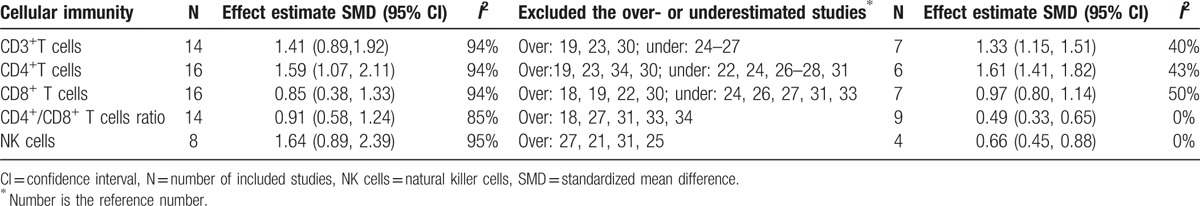
Publication bias and sensitivity analysis.

### 3.8 Sensitivity analysis

There was high statistical heterogeneity between studies in the meta-analysis of CD3^+^T cells, CD4^+^T cells, CD8^+^T cells, CD4^+^/CD8^+^ T cells ratio, and NK cells. These results of the fixed- and random-effect model had good consistency. Therefore, these results were analyzed by random-effects model. After deleting the studies with high weight and significant differences, all results before and after deleting had good consistency. All results showed that the stability was good in this meta-analysis.

## 4 Discussion

In this study, 17 RCTs were finally included. There were 1390 NSCLC (III-IV) patients which included 814 male and 460 female patients between 27 and 78 years of age. The dosage of Aidi injection was 40–100 mL/day and treatment time was 10–28 days. According to the WHO guidelines, meta-analysis showed that Aidi injection plus platinum-based chemotherapy could significantly improve the ORR and DCR of patients with NSCLC. There was no publication bias in any of these studies. Other similar meta-analysis^[35]^ showed that Aidi injection plus TP also could significantly improve the clinical efficiency of patients with NSCLC. This provided indirect evidence for the above conclusions. So far, there was no reliable evidence to confirm the long-term efficacy. Cantharidin and *Astragalus* are important components of Aidi injection. Animal studies^[36–37]^ also showed that cantharidin could significantly inhibit the growth of liver cancer cells and prolong the median survival time of tumor-bearing mice by enhancing the immune function. In vitro studies^[37]^ showed that cantharidin could inhibit the tumor cells proliferation and induce the tumor cells apoptosis. Many studies^[10–13,38–39]^ also showed that *Astragalus*, *Eleutherococcus senticosus* and ginseng had important anti-tumor activity. These results provided evidences for the anti-tumor mechanisms of Aidi injection in NSCLC. In summary, we believe that Aidi injection plus platinum-based chemotherapy can improve the clinical efficacy of patients with NSCLC.

Immune function damage is a serious adverse reaction, including lower anti-tumor and anti-infective immunity induced by platinum-based chemotherapy. This meta-analysis showed that Aidi injection could significantly increase the percentage of CD3^+^T cells, CD4^+^T cells, CD8^+^T cells, and NK cells, and the CD4^+^/CD8^+^ T cells ratio of peripheral blood. But, there was publication bias in the included studies and these results might be overestimated or underestimated. After excluding the over or underestimated studies, meta-analysis showed that the results before and after exclusion had a good consistency. Therefore, these results are scientific and reliable. Zhang et al^[15,40–41]^ found that Aidi injection could reduce the apoptosis rate of peripheral blood lymphocytes and decrease the number of CD4^+^CD25^+^T cells in patients with advanced lung cancer. This study suggests that Aidi injection may enhance cellular immunity through protecting immune cells from apoptosis and reducing Treg cells induced by chemotherapeutics. Xu et al^[42]^ showed that the serum interleukin-6 (IL-6) was increased and the transforming growth factor-β (TGF-β) and tumor necrosis factor-α (TNF-α) were decreased after Aidi injection plus chemotherapy. This study suggests that Aidi injection may enhance cellular immunity by non-specific immune pathway. Many studies^[43–45]^also showed that *Astragalus*, *Eleutherococcus senticosus*, and ginseng were important immunoregulators which could activate the anti-tumor immunity through promoting the tumor immunity-related cytokines and decreasing the chance for the tumor to evade treatment- or metastasis-related cytokines. These studies provided evidences for the mechanisms that Aidi injection could restore the cellular immunity damaged by platinum-based chemotherapy. On the whole, Aidi injection may be an important immune modulator and protector for NSCLC through inspiring the non-specific immune pathway, protecting immune cells from apoptosis, and reducing Treg cells.

## 5 Limitations

There were some limitations in this study. Firstly, Chinese and English databases were retrieved, but not Japanese and Korean databases; all studies were published in China. These might lead to ethnical bias. Secondly, in 17 included trials, only 3 trials described the random allocation method. The allocation concealment was open or implemented in 2 trials and blinding was not described in all of the included trials. These indicated that there were selection bias and implementation bias and therefore led to the overestimation of the efficacy of the treatment group. There was no selective report and other bias was unclear. Thirdly, the long-term efficacy has not been evaluated. This might lead to an inadequate assessment. Fourthly, all immune indicators were detected by different methods in different studies. This might lead to detecting bias or insufficient assessment to cellular immunity. All together, the quality of the included studies is inadequate and the results need to be further confirmed by standardized studies including RCT or real-world studies.

## 6 Conclusions

Aidi injection plus platinum-based chemotherapy can improve the clinical efficacy of patients with NSCLC. Aidi injection could significantly restore the cellular immunity damaged by platinum-based chemotherapy. It may be an important tumor immune modulator and protector for patients with NSCLC treated with chemotherapy. The quality of the included studies is inadequate. The results need to be confirmed by further large sample RCT or real-world studies.

### 6.1 Supporting information

S1 PRISMA Checklist. Preferred Reporting Items for Systematic Reviews and Meta-Analyses (PRISMA) checklist, Figure 1.
